# Supplementary figures and images for: The effect of NASM-based corrective exercises on lumbar lordosis angle and selected muscle activity in women with lower cross syndrome: A randomized clinical trial
Source: PLoS One. 2026 Mar 4;21(3):e0337804. doi: 10.1371/journal.pone.0337804 (PMC12959714; doi:10.1371/journal.pone.0337804)

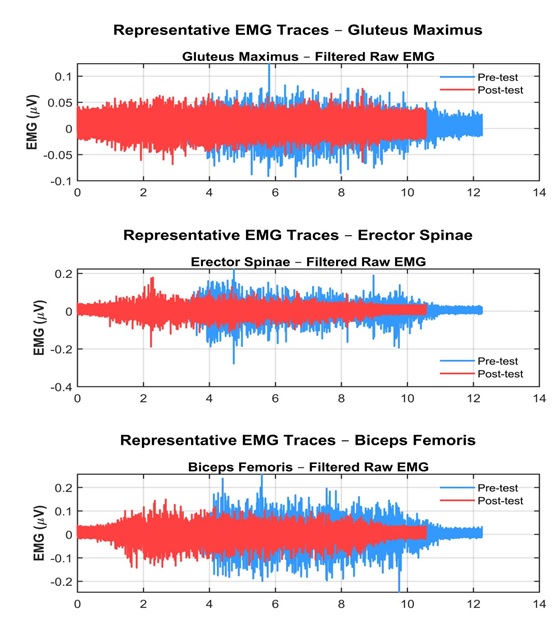

Supplement: S1 Fig — Raw electromyography signals from the gluteus maximus, hamstrings, and erector spinae muscles during the MVIC task pre- and post-intervention. (JPG) [file pone.0337804.s001.jpg]
