# Supplementary material for: The effect of NASM-based corrective exercises on lumbar lordosis angle and selected muscle activity in women with lower cross syndrome: A randomized clinical trial
Source: PLoS One. 2026 Mar 4;21(3):e0337804. doi: 10.1371/journal.pone.0337804 (PMC12959714; doi:10.1371/journal.pone.0337804)
Supplement: S1 File — (DOCX) [file pone.0337804.s002.docx]

# CONSORT 2025 Checklist – Filled Based on Manuscript

Title: The Effect of NASM-based Corrective Exercises on Lumbar Lordosis Angle and Selected Muscle Activity in Women with Lower Cross Syndrome

| Section/Topic | No | CONSORT 2025 Checklist Item Description | Reported on Page No |
| --- | --- | --- | --- |
| Title and Abstract | 1a | Identification as a randomized trial | Title page, Abstract |
|  | 1b | Structured summary of trial design, methods, results, and conclusions | Abstract |
| Open Science | 2 | Trial registration (registry name, ID, URL, and registration date) | The trial was prospectively registered under the clinical trial code IRCT20240805062660N1 (https://irct.behdasht.gov.ir/search/result?query=IRCT20240805062660N1). |
|  | 3 | Where protocol and statistical analysis plan can be accessed | Methods section: Approved by Ethics Committee (ethical code: IR.SSRC.REC.1402.233); not explicitly provided for access, though Supporting Information mentioned (S2 Appendix: Supplementary files) may include it. |
|  | 4 | Where and how individual de-identified participant data and other materials can be accessed | Not applicable / Not stated |
|  | 5a | Sources of funding and role of funders | Funding section: "not supported by any financial funding" |
|  | 5b | Conflicts of interest of authors | Conflict of Interest section: "no conflicts of interest" |
| Introduction | 6 | Scientific background and rationale | Introduction, pages 2–3 |
|  | 7 | Specific objectives | Abstract, Introduction |
| Methods | 8 | Patient or public involvement | Not applicable / Not involved |
|  | 9 | Description of trial design (parallel, allocation ratio, framework) | Methods section: "semi-experimental randomized trial", 2-group design |
|  | 10 | Important protocol changes and reasons | Not applicable (no changes reported) |
|  | 11 | Trial setting and locations | Methods section: "Tehran, Iran; Shahid Beheshti University" |
|  | 12a | Eligibility criteria for participants | Methods section 2.1 |
|  | 12b | Eligibility for sites/interventionists | Not applicable (single-site, same trainers) |
|  | 13 | Intervention and comparator details | Methods sections 2.4, 2.3, and Figure 2 |
|  | 14 | Prespecified outcomes with measurement and timing | Methods section 2.2, 2.3 |
|  | 15 | Definition and assessment of harms | Not specifically assessed or reported |
|  | 16a | Sample size calculation details | Methods section 2.1 (G*Power software, n=24 minimum, recruited 30) |
|  | 16b | Interim analysis and stopping guidelines | Not applicable |
| Randomisation | 17a | Sequence generation method and who generated | Methods section 2.1: "random number generation software" |
|  | 17b | Type of randomisation and restrictions | Equal distribution, no restrictions noted |
| Allocation Concealment | 18 | Allocation concealment mechanism | Not described (likely minimal risk) |
| Implementation | 19 | Who enrolled and assigned participants | Not explicitly stated; by researchers |
| Blinding | 20a | Who was blinded | Not blinded (open-label: exercise vs. no intervention) |
|  | 20b | Blinding method and intervention similarity | Not applicable |
| Statistical Methods | 21a | Methods for comparing groups | Methods section 2.5 |
|  | 21b | Who included in each analysis | Methods/Results: all randomized participants (n=30) |
|  | 21c | Handling of missing data | Not applicable (no dropout reported) |
|  | 21d | Additional/subgroup analyses | Not performed |
| Results | 22a | Participant flow | Methods section 2.1 (Fig 1), Results section, Tables 1-3 |
|  | 22b | Losses/exclusions after randomization | None reported |
|  | 23a | Recruitment and follow-up dates | Methods section 2.1: March–May 2024 |
|  | 23b | Trial end/stop reason | Completed as planned |
|  | 24a | Delivered interventions and adherence | Methods sections 2.3, 2.4 (exclusion if >2 sessions missed; no exclusions) |
|  | 24b | Concomitant care | Not reported (likely not applicable) |
|  | 25 | Baseline characteristics table | Results section, Table 1 |
|  | 26 | Outcomes and estimation | Results section, Table 2 and 3 |
|  | 27 | Harms or unintended events | Not assessed or reported |
|  | 28 | Ancillary/subgroup analyses | Not performed |
| Discussion | 29 | Interpretation and relevance | Discussion section |
|  | 30 | Limitations (bias, generalizability, multiplicity) | Discussion section, partly addressed |
