# Supplementary material for: The effect of NASM-based corrective exercises on lumbar lordosis angle and selected muscle activity in women with lower cross syndrome: A randomized clinical trial
Source: PLoS One. 2026 Mar 4;21(3):e0337804. doi: 10.1371/journal.pone.0337804 (PMC12959714; doi:10.1371/journal.pone.0337804)
Supplement: S2 File — (DOCX) [file pone.0337804.s003.docx]

Faculty of Sports and Health Sciences

Research proposal draft

**Thesis/Dissertation Title: The Effect of Eight Weeks of NASM Exercises on Lordosis Angle, Range of Motion of Spine and Hip, and Electromyography of Selected Muscles in Women with Lower Cross Syndrome**

Student's name and surname: Somayeh Ghaffari

Field of study and specialization: Sports pathology and corrective movements

Request for approval of the topic of the master's thesis Ms Doctoral Thesis (Ph.D)

Professor/Supervisor Profile

| **Row** | **First and last name** | **University-Faculty** | **Educational group** | **Main specialization** | **Academic rank** | **Signature** |
| --- | --- | --- | --- | --- | --- | --- |
|  | **Seyyed Mohammad Hosseini** | **Shahid Beheshti - Sports and Health Sciences** | **Rehabilitation and wellness** | **PhD in Sports Pathology and Corrective Movements** | **Assistant Professor** |  |
|  | **Mehdi Gheitasi** | **Shahid Beheshti - Sports and Health Sciences** | **Rehabilitation and wellness** | **PhD in Sports Pathology and Corrective Movements** | **Assistant Professor** |  |

**Professor/Advisor Professors' Profile**

| **Row** | **First and last name** | **University-Faculty** | **Educational group** | **Main specialization** | **Academic rank** | **Signature** |
| --- | --- | --- | --- | --- | --- | --- |
|  |  |  |  |  |  |  |
|  |  |  |  |  |  |  |

Date of presentation to the group (day/month/year):

Date of approval in the group (day/month/year):

Anticipated date for thesis/dissertation defense (day/month/year):

Persian and Latin keywords (5-7 sentences, each sentence should not exceed two parts)

| **Keywords:** Corrective exercises, Range of motion, Lower cross syndrome, Electromyography. |
| --- |

**Definition of conceptual-operational keywords**

| **Lower Crossed Syndrome**  Conceptual definition: It is a musculoskeletal condition characterized by a pattern of muscle weakness and stiffness (muscle imbalance) present in the central region of the body. ^[[1]](#footnote-1)^  Operational definition: In the present study, the upper lumbar lordosis angle of 50 degrees will be considered flexible using a ruler.  **Range of motion**  Conceptual definition: Range of motion is the total movement that a joint is allowed to move through from the starting position to the end of the movement.  Operational definition: In the present study, the modified Schober test, hip flexion, and hip extension using a goniometer will be used to assess the range of motion of lumbar flexion.  **Electrical activity of muscles**  Conceptual definition: Electrical activity of muscles reflects force production in the muscle.  Operational definition: In the present study, maximal voluntary isometric electrical activity and the activation sequence of selected muscles will be examined using an electromyography device. The amount of muscle activity will be measured using the normalization of maximal voluntary contraction (MVIC). |
| --- |

**1) Introduction and statement of the problem**

| Lower Crossed Syndrome (LCS) is a musculoskeletal condition characterized by a pattern of muscle weakness and stiffness (muscle imbalance) and a crisscross pattern in the sagittal plane. Lumbar-pelvic movement disorders may cause this condition. Lower crossed syndrome involves weakness of the trunk muscles, including the rectus abdominis, internal oblique, external oblique, and transverse abdominis muscles, along with weakness of the gluteus maximus, middle buttocks, and small buttocks, which are inhibited, resulting in their function being replaced by superficial muscles. For example, the hamstring muscles compensate for weakness caused by the gluteus maximus muscle. Additionally, overactivity and tightness of the spinal extensors, including the spinal erector muscles, multifidus, quadratus lumborum, and latissimus dorsi, as well as the hip flexors, adductor muscles, and iliotibial band, are associated with this disorder.^[[2]](#footnote-2)^^[[3]](#footnote-3)^^[[4]](#footnote-4)^^[[5]](#footnote-5)^^[[6]](#footnote-6)^^[[7]](#footnote-7)^^[[8]](#footnote-8)^^[[9]](#footnote-9)^^[[10]](#footnote-10)^^[[11]](#footnote-11)^^[[12]](#footnote-12)^^[[13]](#footnote-13)^^[[14]](#footnote-14)^^[[15]](#footnote-15)^  Muscle imbalance caused by lower crossed syndrome leads to joint dysfunction (ligament strain and increased pressure on the vertebrae, especially in the lower back, L4-L5 and L5-S1, sacroiliac joint, and hip joint), resulting in back, hip, and knee pain. It also causes specific postural changes such as anterior pelvic rotation, increased lumbar lordosis, and knee hyperextension. Janda defines muscle imbalance as a disturbed relationship between muscles prone to tightness or shortening and muscles prone to inhibition. The consequences of muscle imbalance in the lower extremities significantly impact not only the biomechanics of the hip joints but also the knee joints, ankle joints, shoulder joints, and other joints of the spine. Consequently, increased thoracic kyphosis and increased cervical lordosis are also complications of this syndrome. To better understand the relationship between abnormalities and the interrelationship between the musculoskeletal and nervous systems, Janda pointed out that any disorder or defect in any of the muscles and joints in the body also affects the quality and function of other joints and muscles. Changes in the joints and muscles in one area following a disorder in one position are transmitted to other areas of the body through a chain reaction and affect adjacent joints and muscles.^[[16]](#footnote-16)^  Lower cross syndrome can develop as a result of various situations, including prolonged and repetitive activities. Conversely, inactivity can negatively impact body mechanics. For example, chronic postural pain syndrome or prolonged sitting at work and poor posture can lead to this condition. As a result, lower crossed syndrome can cause back problems (such as back pain) due to postural imbalances. Janda states that due to prolonged, fixed postures, such as sitting at a desk all day, the hip flexors become shortened or tight. Therefore, the brain automatically begins to shut down or inhibit the gluteal muscles on the opposite side. Currently, the imbalance pattern causes increased lumbar lordosis due to increased anterior pelvic tilt and hip flexion, resulting in overactivity of the hip flexors to compensate for abdominal weakness. The gluteus maximus is inhibited as an antagonist of the hip flexors, reducing the quality of hip extension, so the spinal erectors and hamstrings must be overactive and eventually tightened to provide the necessary hip extension instead of the gluteus maximus.  People in the late teenage age group up to 40-year-olds are very active in their daily lives and are exposed to various stresses. Unlike older individuals, age-related changes in this age group are minimal. Burton AK (1996) showed that the prevalence of low back pain in young adolescents is similar to that in adults. Also, GT Jones et al. (2005) show in their study that the lifetime prevalence reaches 70 to 80% by the age of 20. Another study concluded that 85% of back pain is caused by muscle imbalances, usually due to long-term postural defects called lower cross syndrome. According to studies, women had a higher prevalence of lower crossed syndrome compared to men in the same age group. It was also found that there was no significant difference in left lumbar spine stiffness between boys and girls of the same age. However, there was a significant difference in the length of the spinal erectors, abdominal muscle strength, maximum bilateral gluteal strength, and right lumbar spine length between boys and girls of the same age, with girls experiencing more weakening of these muscles.  Changes in the natural curve of the back are related to several factors, and muscle imbalance can be considered the main cause of these abnormalities in the lumbo-pelvic area. With impaired muscle function in this area, the spine, which is maintained and supported by these muscles, is put under pressure, resulting in changes in the curve of the back, and back pain is one of its consequences. The use of corrective exercises reduces structural abnormalities by improving and promoting muscle balance and, consequently, improving the function of the human motor structure.  The consequences of poor posture, such as increased lumbar curvature, negatively impact physical, psychological, economic, and social dimensions. Inappropriate body movements or prolonged postures in individuals cause postural abnormalities and, as a result, lead to disorders in various body systems, including the musculoskeletal system. There are numerous complications, such as pain, reduced range of motion, and deformity. Research has shown that if the body is in an unfavorable posture (postural abnormalities) for a long time, some muscles become stretched and some become shortened, adapting to this situation. This adaptation occurs in a way that causes muscle contraction and stiffness in the shortened muscles and weakness and stretching in the muscles on the opposite side. In this regard, research results show that changes in muscle structures and changes in muscle recruitment cause changes in the dynamics of the pelvic girdle and disrupt the tension-contraction relationships and muscle activation time. Furthermore, according to the concept of muscle slinging and the interconnectedness of connecting muscles in kinetic chains, dysfunction of muscle chains in the body leads to suboptimal load transfer and joint movement control strategies, which in turn cause changes in range of motion and excessive forces on the lumbar spine. It has also been proven that core stability plays a key role in proper muscle function. Studies have shown that the core muscles contract before any movement in the limbs to provide a stable base to support the movement, independent of the direction of movement. Any disorder or weakness in the activity of these muscles disrupts the muscular forces of the peripheral parts, and efficient movements cannot be witnessed.  The movements of the hips, pelvis, and lumbar spine are closely interconnected and perfectly coordinated. The interactions between the hips and thighs are commonly referred to as the pelvic-femoral rhythm, and between the lumbar spine and pelvis as the lumbopelvic rhythm. Abnormal and asynchronous waist-pelvis-hip patterns lead to excessive loading of the waist and hip joints during trunk movements. For example, pelvic rotation is increased during active hip flexion in individuals with femoral impingement or during trunk flexion, leading to increased loading of the spine. Limited range of motion of the thigh (ROM) contributes to defective lumbopelvic or pelvic-femoral rhythm and resulting spinal misalignment due to muscle stiffness and other joint tissues. Muscle tightness increases lumbar hyperlordosis in standing.  Hip extension is required for a variety of functional activities. Lack of adequate hip extension leads to gait impairment and increased impact forces on the joints during activity. Prone thigh extension is a common and widely accepted test for measuring lumbopelvic movement patterns. The importance of the prone hip extension is that the movement patterns during this test are used to simulate those used in functional movement patterns such as walking. It is hypothesized that changes in this pattern can reduce lumbopelvic stability during gait and place abnormal stress on the lumbar lordosis, leading to low back pain. Good reliability has been reported for the prone hip extension in detecting lumbar spine deviation from the hypothesized line of gravity. Coordination between muscles in the lumbopelvic region is thought to maintain the pelvic position in a normal and balanced position during lower limb or trunk movement. In this regard, it has been shown that overactivity of the spinal erector muscles and inhibition or delay in gluteus maximus activity cause anterior pelvic tilt and increased lumbar lordosis, especially in individuals with lower crossed syndrome. It has been theorized that muscle activation during extension should occur in the following order: Right leg extension: right gluteus maximus, right hamstring, left lumbar erector spinae, right lumbar erector spinae. As a result, the delay in the recruitment of the gluteus maximus results in an inefficient pattern of muscle activation. The weakest recruitment and activation pattern in hip extension occurs when the ipsilateral spinal erectors initiate the movement. As a result, the activation of the gluteus maximus muscle is delayed, and hip extension is achieved by the activation of the hamstrings, anterior pelvic tilt, and lumbar hyperlordosis.^[[17]](#footnote-17)^  In the past, there were various methods of correcting abnormalities, and they were performed separately by applying separate stretching and strength training methods to different abnormalities caused by different syndromes. However, along with new scientific advances, new exercises have been developed to increase such abnormalities. Recently, the National Academy of Sports Medicine (NASM) in the United States has introduced new corrective exercises to restore muscle imbalances, which include four phases of inhibition, stretching, activation, and coordination. In this protocol, it is recommended that instead of simply stretching the shortened or tight muscle, it is better to first perform resistance exercises and then stretching exercises on the muscle. In the myofascial release technique, the individual himself creates an inhibitory response in the muscle spindle and reduces the activity of the gamma circuit through continuous pressure with a specific intensity, rate, and duration, stimulating the aforementioned receptors. Also, for underactive muscles, instead of simply strengthening them, it is better to use cohesion exercises at the end.^[[18]](#footnote-18)^^[[19]](#footnote-19)^  Since optimal and desirable neuromuscular efficiency for maintaining dynamic stability is established by the appropriate combination of correct postural alignment and stabilizing strength, any defect in the alignment of the body and the lumbopelvic girdle can, through changes in the relationship of muscle force pairs and changes in mechanical performance, cause changes in different parts or even the work of specific components, systems, and organs related to the locomotor system. Therefore, following the functional and structural changes of the anterior pelvic tilt and increased lumbar curvature in people with lower crossed syndrome, the reception of stimuli by peripheral receptors and their conversion into nerve signals and transmission through afferent pathways to the central nervous system changes, and in this way, the entire sensory-motor system may be disrupted. As a result, instability in the lumbopelvic girdle will occur.  Considering the above-mentioned contents, given that corrective exercises by NASM are a non-invasive method for improving rehabilitation, enhancing quality of life, and reducing health, economic, and social costs. Therefore, despite the existing ambiguities and contradictions, the purpose of the present study is to determine the effectiveness of eight weeks of corrective exercises by NASM on lordosis angle, spine and hip range of motion, and selected muscle activity in women with lower crossed syndrome. |
| --- |

The necessity and importance of research.

| The lumbar spine is one of the most important parts of the spine, which is of particular importance due to its unique position and direct connection to the pelvis. Any increase or decrease in the amount of lumbar arch affects the balance of the entire body posture and leads to various abnormalities and disorders in the lumbopelvic area. Lower cross syndrome, as one of the most important disorders of the body, brings about changes in the lumbar arch. Also, given the importance of the issue the abnormality, considering the numerous complications and consequences caused by it and the impact of lower cross syndrome on adjacent joints, such as back pain and anterior knee pain, correction of abnormalities should be on the agenda. Also, in order to reduce the treatment costs of possible future problems of people with lower crossed syndrome, as well as to improve the posture of people and increase muscle efficiency, the use of non-invasive and low-cost treatment methods is considered important. Therefore, given the limited studies conducted on the correction of lower crossed syndrome abnormalities, according to the searches conducted by the researcher, as well as the lack of research that uses coherent corrective exercises by NASM, the present study will be conducted to improve the deformity of lower crossed syndrome. |
| --- |

**Overall goal.**

| The present study aimed to determine the effectiveness of corrective exercises by NASM, focusing on lordosis angle, spinal range of motion, core muscle endurance, and electrical activity of selected lumbopelvic and lower extremity muscles in individuals with lower crossed syndrome. |
| --- |

Specific goals.

| 1. Determining the effectiveness of corrective exercises by NASM on lordosis angle in people with lower crossed syndrome. 2. Determining the effectiveness of corrective exercises by NASM on the spinal range of motion in people with lower crossed syndrome. 3. Determining the effectiveness of corrective exercises by NASM on hip range of motion in people with lower crossed syndrome. 4. Determining the effectiveness of corrective exercises by NASM on the electrical activity of selected lumbopelvic and lower limb muscles during the hip hyperextension task in individuals with lower crossed syndrome. |
| --- |

**Hypotheses.**

| 1. Corrective exercises by NASM have a significant effect on the lordosis angle in people with lower crossed syndrome. 2. Corrective exercises by NASM have a significant effect on the spinal range of motion in people with lower crossed syndrome. 3. Corrective exercises NASM, have a significant effect on hip range of motion in people with lower crossed syndrome. 4. Corrective exercises have a significant effect on the electrical activity of selected lumbopelvic and lower limb muscles during the hip hyperextension task in individuals with lower crossed syndrome. |
| --- |

**6) Research methodology**

**1-6) Type of research**

| The present study is a randomized clinical trial that was conducted after receiving the code from the Ethics Committee in Biological Research in the Iranian Clinical Trial System. IRCT will be prospectively enrolled. The present study will include two experimental and a control groups. The individual shown in Figs. 1, 2, and 3, as well as in the Corrective Exercise Protocol NASM (Table 1), has provided written informed consent (as outlined in the PLOS consent form) for the publication of their images alongside this article. |
| --- |

**2-6) Subjects (subject characteristics, sampling method, inclusion criteria, and exclusion criteria)**

| **Statistical population**  The statistical population of the present study will consist of inactive female students in Tehran. The sample size was determined by the GPower software (G*Power software, version 3.1.9.7, Heinrich-Heine University, Düsseldorf, Germany (Power*G Windows) will be determined. According to information from past studies, with a confidence factor of 0.95, power of the test 0.95, and effect size 0.40, for each of the research groups, at least 12 subjects will be required, which, taking into account the possible dropout, will be considered for participation in each group by 15 subjects. Finally, the subjects of the present study will be 30 girls with lower crossed syndrome, who were selected using a purposive sampling method and randomly generated using random number generator software (Random Number Generator), and will be divided into two experimental groups and a control group. These individuals will be selected through screening in general physical education classes at the university.  **Criteria Login**  _Women with lower crossed syndrome  _ Past lack of participation in regular sports and aerobic activity for at least three months  _ Age range 18-30 years old  _ Upper lordosis angle 93.58 degrees (using the Judas scale) (36)  _ BMI 20 to 25^[[20]](#footnote-20)^  **Research exclusion criteria**  _ Having back pain with orthopedic causes  _ History of regular sports activity  _ Having other postural abnormalities that affect the research process, such as hyperkyphosis, scoliosis  _ Injury and pain during the research process  _ Absence of more than two training sessions |
| --- |

**3-6 (Research plan)**

| Age range of subjects: The age range will be 18 to 30 years. 15 people will be randomly assigned as the control group and 15 people as the experimental group (NASM corrective exercise protocol). |
| --- |

**4-6) Research implementation method**

| After selecting the subjects, the necessary information regarding the purpose and method of conducting this research and the points that must be observed by the subjects to participate in this research will be provided to the subjects orally. Then, a consent form will be collected from the subjects to declare their readiness to participate in this research. The individual shown in Figs. 1, 2, and 3, as well as in the Corrective Exercise Protocol NASM (Table 1), has provided written informed consent (as outlined in the PLOS consent form) for the publication of their images alongside this article. It will also be explained to the subjects that at any time during the research process, if they do not wish to continue their cooperation, they can withdraw. They can opt out. Then, to match and select the subject, Subject demographic characteristics plural form. This form will contain information related to the feature. Personal data (height, weight, age, pain, and history of sports activity) and medical history will be collected. Participation in the study will be completed by the subjects, and a file will be created for them. Field tests will include lumbar lordosis angle using the Judas scale, spinal range of motion using the modified Schober test, and hip flexion and extension range of motion using a goniometer. Also, the electrical activity of the gluteus maximus (right), hamstrings (right), and erector spinae (left) muscles will be recorded during thigh hyperextension using an electromyography device in the laboratory of the Physical Education and Sports Sciences Research Institute. All tests will be performed within one week before the start of the exercises as a pre-test and one week after the end of the exercises as a post-test. ­­­­­ |
| --- |

**5-6) Data collection method**

| Measuring the lordosis angle  The flexible ruler with the brand Idio, made in Thailand, is a rod that can be bent and straightened, easily takes shape, and is graduated. The validity of this tool compared to radiography (r = 0.91) is determined (reference). To measure lumbar curvature, the subject will stand in a completely comfortable and natural position with bare feet on a cardboard with the location of the feet marked. The subject will be asked to spread their legs shoulder-width apart, look straight ahead, and be completely relaxed. Then, the examiner will stand behind the subject to find the reference points. These points are: Spinous process of the twelfth dorsal vertebra (T12) as the starting point of the arch and the second sacral vertebra (S2) as the end of the arc (2). Using an oil pen, the skin will be marked. Then, the ruler will be placed on the desired points, and by pressing the ruler against the lumbar curve, a curvature will be created in the ruler according to the lumbar curve. Then the marked points will be marked on the ruler and the ruler will be placed on the paper without any changes and in the least time. Then a curved pen will be drawn on the paper. After removing the ruler from the paper, the two marked points T12 and S2 will be connected by a straight line. Measure the length of the line and label it with the letter L. In the next step, from the deepest point of the arch, a linear curve will be drawn on the L line, and the depth of the arch (H) will be measured. Finally, the lordosis angle will be measured using the following formula: (37, 38). ^[[21]](#footnote-21)^^[[22]](#footnote-22)^  θ=4[ARCtag^(2𝐻/𝐿)^]  **Assessing spinal range of motion**  Modified Schober's double test, which is a valid and reliable method for measuring the range of motion of the lower back in trunk flexion and extension, will be used in this study. In this method, the midpoint of the anatomical location of the two posterior superior iliac spines (ASIS) will be determined as the first point, and 15 cm above that will be determined as the second point. The increase in distance after bending forward and the decrease in distance after bending backward will be the amount of flexibility of the back in bending forward and backward. The patient's pelvis will be held and immobilized, and the patient will be asked to bend forward as much as possible. The changes made in the 15 cm distance will be defined as the range of motion of bending forward. For the modified Schober test, the average validity (r=0.67) and external (r=0.91), and internal (r=0.95) reliability have been reported.^[[23]](#footnote-23)^^[[24]](#footnote-24)^  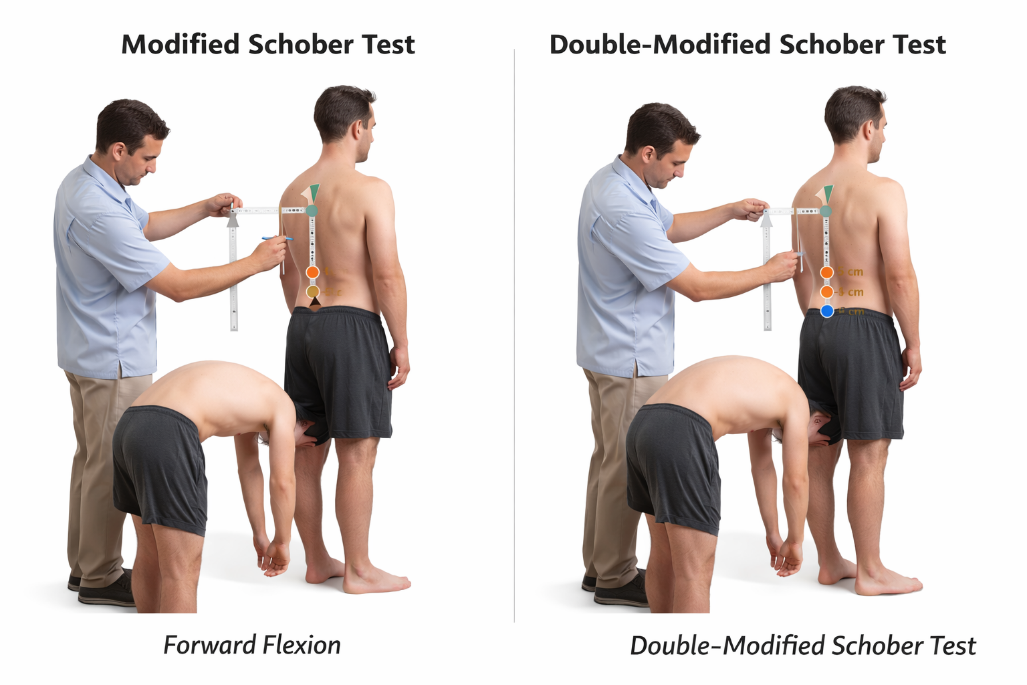  **Shape1. Modified Schober's Double Test**  **Assessing hip flexion and extension range of motion**  **Hip flexion range of motion**  For this purpose, the subject will be asked to lie in an open-chested position. The knee and thigh of the non-test leg will be placed in an extended position (without abduction or adduction), and the desired leg will be used to perform hip flexion with the knee bent. The center of the goniometer will be placed on the greater trochanter of the thigh, the fixed arm will be parallel to the lateral bisector of the pelvis and the examination table, and the movable arm will be parallel to the lateral bisector of the femur towards the lateral epicondyle.  **Hip extension range of motion**  To measure hip extension range of motion, the subject will be placed in a prone position and will perform hip extension with the tested limb with the knee straight. The goniometer placement for measuring hip range of motion will be similar to the flexion test.  **Recording the electrical activity of selected muscles**  With attention to the transfer force throughout the pelvis to the face, the crossed chain of muscles (hamstrings) - gluteus maximus - muscles on the right side of the lumbar spine and the opposite side will be involved. This is similar to daily movements where synergy occurs. The electrical activity of these muscles will be recorded. For the registration of electrical activity, the right lumbar spine (left side) and the right side of the gluteus maximus, as well as the hamstrings (biceps femoris on the right side), will be measured using the Wireless Mayon EasyEMG device, made in Switzerland, with a sampling frequency of 2000 Hz. To achieve this, the first step will be to prepare the skin of the subjects based on the European Electromyography SENIAM protocol. This will involve accurately determining the electrode locations. Excess hair will be removed using a Gillette razor, and the skin will be thoroughly cleaned with cotton and alcohol to eliminate dead skin cells and other factors that may influence skin resistance. The electrode locations will be determined based on anatomical landmarks and the isometric contraction of the muscles to identify the bulk muscular usage. Superficial electrodes will be placed parallel to the muscle fibers, following standard methods as described below for the desired muscle locations.   1. Muscle Right Maker Column Nut I see (Longissimus) Side Left: Three Centimeter Appendix Shocking Nut Third Camry (43, 44).^[[25]](#footnote-25)^ 2. Muscle Cerini Big Right: One Third Foreign Between Crown Khasra and Trochanter Big Thigh (43-45). 3. Muscle Biceps curl Right: In Middle Route Linear Prominence Living room Up to Hole Popliteal (43, 44).^[[26]](#footnote-26)^   After installing the electrodes on the desired muscles, the electrodes will be fixed with an anti-allergic adhesive and bandage to prevent the electrodes from moving on the skin and causing noise. Also, to prevent noise, the wires will be collected and tied to the person's body using a bandage. Participants will be placed in different test positions, the method of implementation of which is as follows:   \| \| 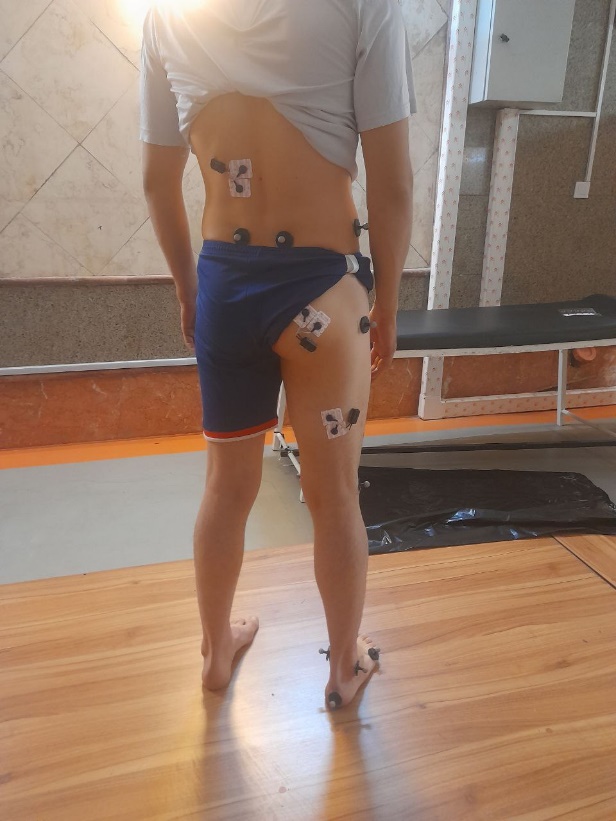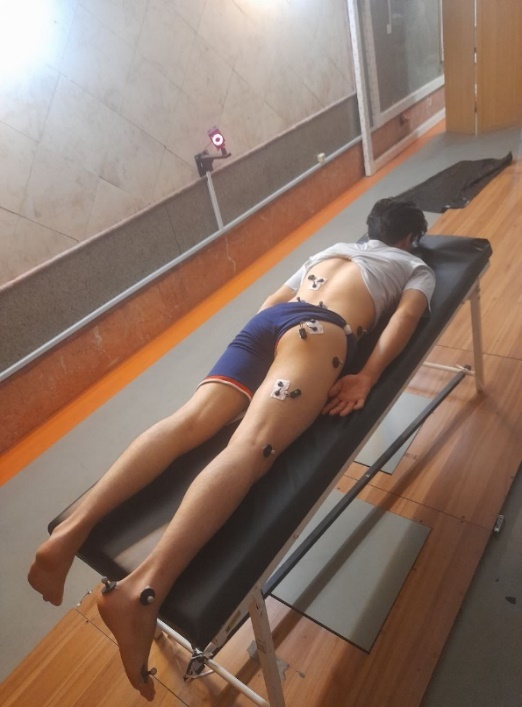 \| \| --- \| \| \| --- \| --- \| \| **Shape 2. Placement of surface electrodes parallel to the muscle fibers of the erector spinae, biceps femoris, and gluteus maximus muscles.** \|   **Hip hyperextension**  In this task, the electrical activity of the gluteus maximus, hamstrings, and erector spinae muscles will be recorded. To perform this test, the subject will lie facedown on a table with the body straight and extended, with the toes dangling over the edge of the table. The opposite ankle will be strapped to the table, and the subject will then perform a hip hyperextension movement (approximately six inches off the table). At this point, the subject will hold the position isometrically for five seconds, and this process will be repeated three times with a 60-second rest between each repetition.   \| 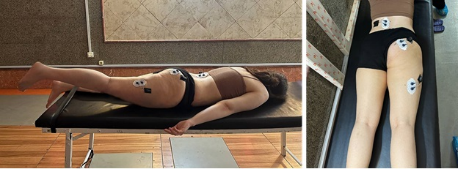 \| \| --- \| \| **Shape3. Hip hyperextension** \|   Calculating maximum voluntary contraction and the time of onset of muscle activity: In order to normalize electromyographic data, the method MICV was used. In this way, after recording the activity of each muscle under test, the middle 3 seconds will be selected. Also, to calculate the onset of muscle activity, in all tasks, the waves are first rectified, and three times the standard deviation of the electrical activity of the muscles in the background line will be considered as the threshold for the onset of activity. The time of onset of muscle activity will be the point when the muscle activity reaches the threshold and remains above the threshold level for at least 25 milliseconds. Finally, to calculate MVIC, to perform calculations, the files recorded in the Megawin program will first be converted to ASCII format and examined in the MATLAB program. The electromyographic signals will be passed through an 11 Hz filter in the program written by an electronics expert in the MATLAB environment, and then the items mentioned in the signals will be taken into account to detect muscle activity.^[[27]](#footnote-27)^  After measuring the subjects' characteristics (height and weight) and initial assessments (lordosis angle, spinal range of motion, core muscle endurance, and electrical activity of selected muscles), the subjects will participate in this study in two control and experimental groups for eight weeks. The subjects in the experimental group will participate in the exercise program. NASM will attend three sessions per week for eight weeks, each session lasting 60 minutes, and the control group will continue with their normal daily activities.  **Corrective exercise protocol NASM (Table 1)**  This protocol includes four technique stages: inhibit, lengthen, activate, and integrate. Participants were asked to perform the main exercise program after a five-minute general warm-up. The program included resistance, stretching, activation, and coordination exercises.  Inhibition and lengthening exercises were performed during the first two weeks. In the second two weeks, the same exercises continued, but lengthening was applied to the muscles that had been inhibited. These exercises were considered the main part of the program, and activation exercises were added.  In the third two weeks, muscle activation exercises made up the majority of the session, and the previous exercises were continued if necessary. Finally, in the fourth two weeks, integration exercises were added to the previous routine.  **Technique – Inhibit:**  At this stage, to release tension or reduce overactivity in the neuromyofascial system, foam rolling (hard type) was used. This increases pressure on soft tissue structures and reaches deeper layers of the fascia. In this protocol, participants foam rolled the desired area for 30 seconds.(35)  **Technique – Lengthening:**  This method aimed to increase tissue elasticity, muscle length, and range of motion. Static stretching was performed at the first point of resistance and held for 10 to 30 seconds.(50)  **Technique – Activation:**  Activation exercises were used to increase neuromuscular activity. Each exercise was performed for 10 to 15 repetitions, with each repetition including a 1 to 2-second isometric hold at the end of the range of motion, followed by a 4-second eccentric contraction.(50)  **Technique – Integrate:**  This technique focused on neuromuscular re-education and coordination through functional movements. Dynamic, total-body exercises were used to promote muscle synergy, improve stabilization, and enhance movement efficiency.  **Table 1.Week 1 and 2 training schedule**   \| Exercise 1:  Self Myofascial Release of the Hip Flexor Muscles2 to 3 sets of the movement are performed, holding the pressure of the foam roller on painful points for 30 to 90 seconds. \| 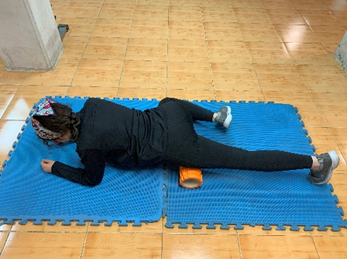 \| \| \| --- \| --- \| --- \| \| Exercise 2:  Self Myofascial release of the latissimus dorsi muscle is performed in 2 to 3 sets, with pressure from the foam roller held on painful points for 30 to 90 seconds. \| 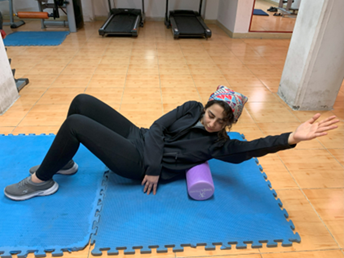 \| \| \| Exercise 3:  Self Myofascial release of the hamstring muscle is performed in 2 to 3 sets, holding the pressure of the roller at painful points for 30 to 90 seconds. \| 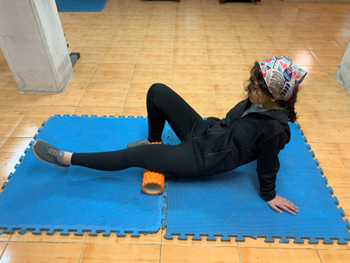 \| \| \| Exercise 4:  for Self-myofascial release of the lumbar erector spine is performed in 2 to 3 sets, with a pressure hold of the foam roller on painful points for 30 to 90 seconds. \| 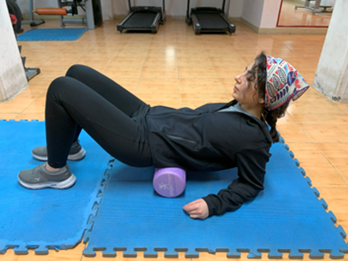 \| \| \| Exercise 5:  for the release of the quadratus lumborum muscle is performed in 2 to 3 sets, with a pressure hold of the foam roller on with a pressure hold of the foam roller on painful points for 30 to 90 seconds. \| \| 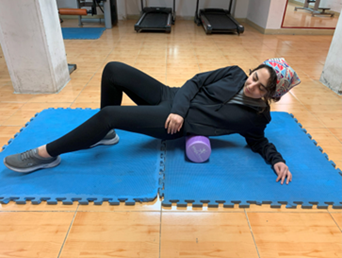 \| \| Exercise 6:  Static stretching of the lumbar  erector spine is performed in 2 to 3 sets,  with each stretch lasting 10 to 20 seconds. \| \| 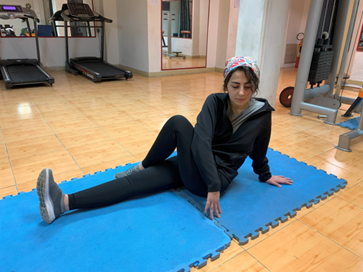 \| \| Exercise 7:  Static stretching of the hip flexor muscles is performed in 2 to 3 sets.  The movement lasts for 10 to 20 seconds. The leg that is positioned back is raised and bent towards the opposite side. \| \| 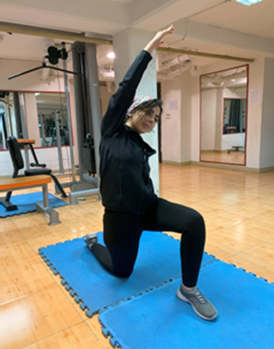 \| \| Exercise 8:  Static stretch of the latissimus dorsi muscle is performed in 2 to 3 sets. The stretch is held for 10 to 20 seconds. \| \| 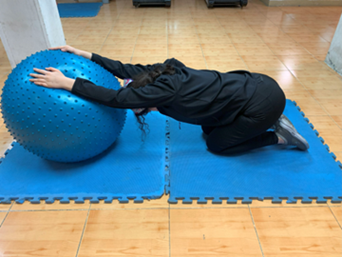 \| \| Exercise 9:  Static stretching of the hamstring muscle is performed in 2 to 3 sets. The stretch is held for 10 to 20 seconds. \| \| 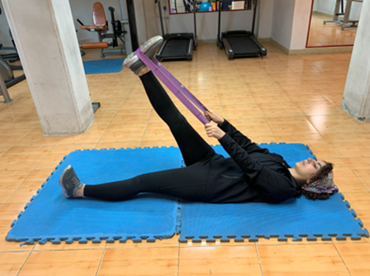 \| \| Exercise 10:  Static stretching of the hip flexor muscles is performed in 2 to 3 sets, with each stretch lasting 10 to 15 seconds \| \| 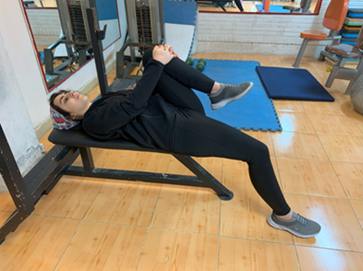 \| \| Exercise 11:  Neuromuscular stretching (PNF ) of the hamstring muscles is performed in 2 to 3 sets. \| \| 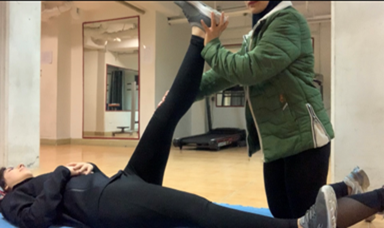 \|   **Week 3 and 4 training schedule**  Self-myofascial release exercises for the hip flexor muscles, self-myofascial release for the latissimus dorsi muscle, self-myofascial release for the hamstring muscle, self-myofascial release for the lumbar erector spinae muscle, self-myofascial release for the quadratus lumborum muscle, each performed for three sets of 20 to 25 seconds.   \| Exercise 1:  Static stretching of the lumbar erector muscles, the cat movement is performed in 3 to 4 sets, with 20 seconds of stretching executed. \| 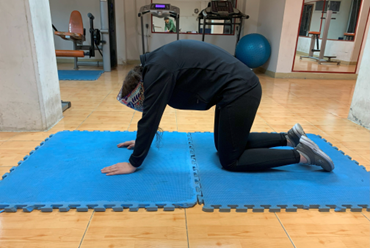 \| \| --- \| --- \| \| Exercise 2:  Stretching the lumbar erector spine muscles can be performed either statically or dynamically. It is done in three sets of 20 seconds or three sets of 20 repetitions (rocking motion). \| 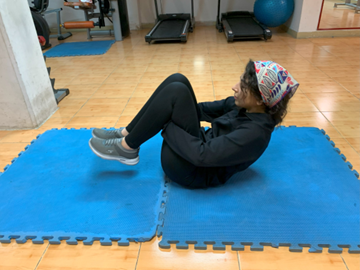 \| \| Exercise 3:  Static stretching of the flexor muscles is performed in three sets of 20 to 25 seconds. Note: do not increase the arch of the lower back. \| 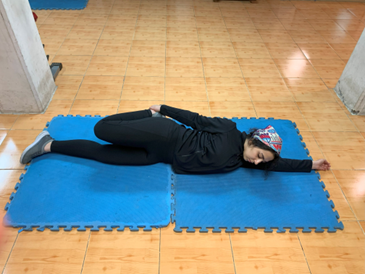 \| \| Exercise 4:  Static stretch of the latissimus dorsi muscle is performed for 20 to 25 seconds in three sets. \| 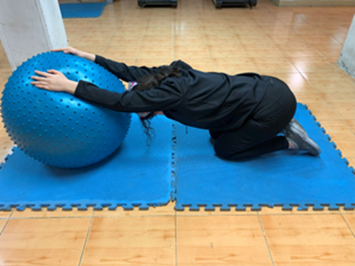 \| \| Exercise 10:  Crunch on the ball This exercise is performed to strengthen the abdominal muscles.  The crunch movement on the ball is done If the person cannot perform it on the ball and loses balance, they can do it on the ground. \| 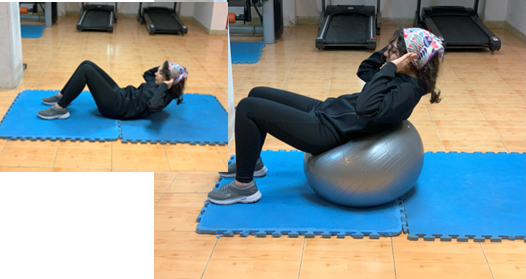 \| \| Exercise 11:  The bridge on the ball is performed to strengthen the gluteus maximus muscles.  If the individual is unable to perform it with the ball, it is done on the ground. Three sets of 20 seconds are performed. \| 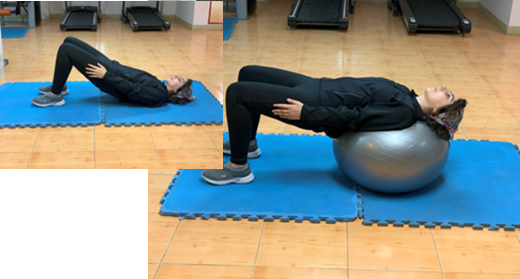 \|   **Week 5 and 6 training schedule**   \| Exercise 1:  Dynamic stretching of the hip flexor muscles is performed in 3 sets of 15 to 20 repetitions.  To start, the front leg should be more than 90 degrees and the back leg at 90 degrees, and the individual performs the stretch dynamically by moving forward and backward. \| 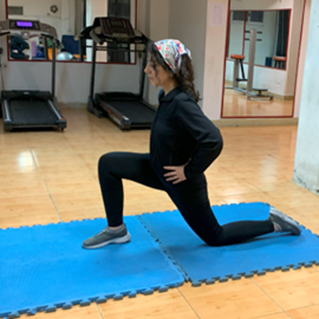 \| \| --- \| --- \| \| Exercise 2:  Dynamic stretching of the hamstring muscle is performed for 15 to 20 repetitions. ( 3sets) The leg is stretched in three directions: middle, inside, and outside. \| 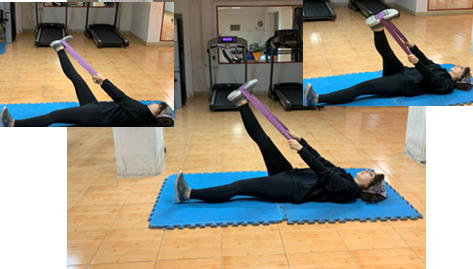 \| \| Exercise 3:  Dynamic stretching of the erector spine muscles is performed for 10 repetitions (3 sets), and at the end of each set, the stretch is held forward for 20 seconds. \| 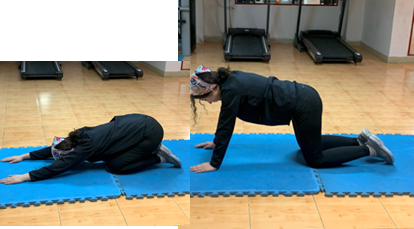 \| \| Exercise 4:  Crunch on a ball or crunch on the ground is prformed in 3 sets of 15 to 20 repetitions. \| \| \| Exercise 5:  Modified plank )stability exercise(,three sets of 15 to 20 seconds, which can be done with a ball or for those who cannot perform it on the ground. \| 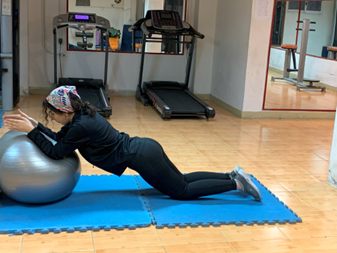 \| \| Exercise 6:  Bridge on the ball is performed for three sets of 15 to 20 seconds to strengthen the gluteus maximus muscles. \| 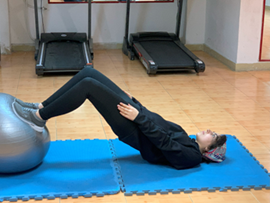 \| \| Exercise 7:  ( Dead bug ) Strengthening the abdominal muscles, with 10 to 15 repetitions. The opposite hands and feet are opened and when they are opened, they do not touch the ground, and then we return to the initial position and repeat again. \| 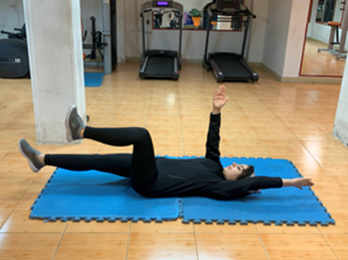 \| \| Exercise 8:  Strengthening the gluteus maximus, three sets of 10 to 15 repetitions or holding the leg up for 15 to 20 seconds. \| 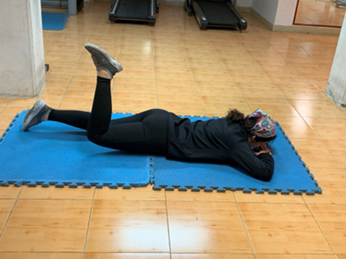 \|   **Week7 and 8 training schedule**     \| Exercise 1:  Dynamic stretching of the erector spinae muscles, 4 sets of 15 repetitions. \| \| 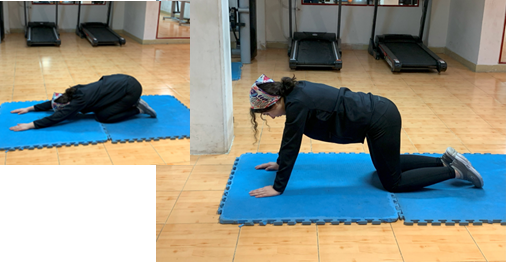 \| \| --- \| --- \| --- \| \| Exercise 2:  Dynamic stretching of the hip flexor muscles is performed in 3 sets of 20 repetitions. \| \| 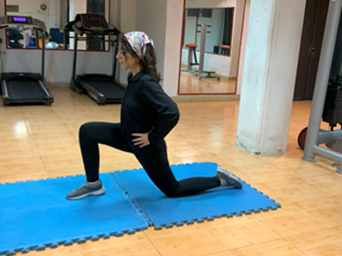 \| \| Exercise 3: Crunches can be done on a ball or on the ground, three sets of 10 repetitions. \| \| \| \| Exercise 4:  Bridge on the ball to strengthen the gluteus maximus muscles, performed in 3 sets of 20 seconds. \| 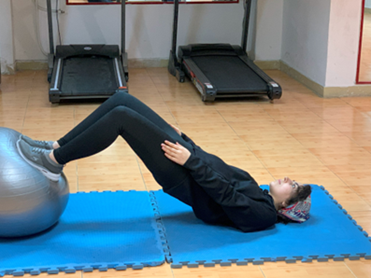 \| \| \| Exercise 5: The Dead bug movement is performed in 4 sets, 10 to 15 repetitions. \| \| \| \| Exercise 6:  The bird dog fetches for 15 to 20 seconds or is repeated 15 to 20 times in each set. \| 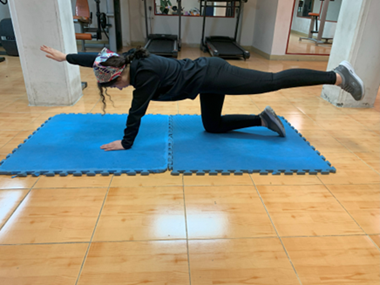 \| \| \| Exercise 7:  Strengthening the gluteus maximus is held up for 3 sets of 3 to 20 seconds  . \| 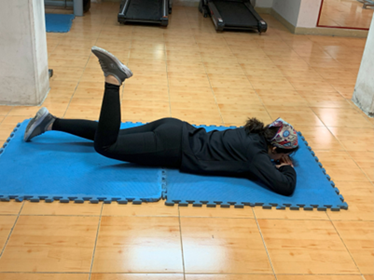 \| \| \| Exercise 8: Modified plank is performed for 4 sets of 20 seconds each. \| \| \| \| Exercise 9:  Dynamic cohesive squat movement with a ball against the wall along with an overhead press, 3 to 4 sets of 20 repetitions. \| 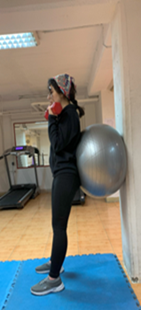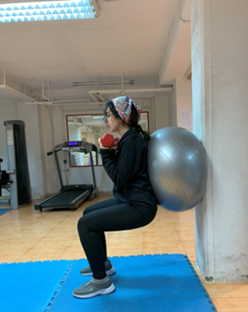 \| \| |
| --- | --- | --- | --- | --- | --- | --- | --- | --- | --- | --- | --- | --- | --- | --- | --- | --- | --- | --- | --- | --- | --- | --- | --- | --- | --- | --- | --- | --- | --- | --- | --- | --- | --- | --- | --- | --- | --- | --- | --- | --- | --- | --- | --- | --- | --- | --- | --- | --- | --- | --- | --- | --- | --- | --- | --- | --- | --- | --- | --- | --- | --- | --- | --- | --- | --- | --- | --- | --- | --- | --- | --- | --- | --- | --- | --- | --- | --- | --- | --- | --- | --- | --- | --- | --- | --- | --- | --- | --- | --- | --- | --- | --- | --- |

**Method Statistical data**

| To determine the normality of the data, the Shapiro-Wilk test and the Levene test will be used to examine the homogeneity of variance. Mixed analysis of variance will be used to assess the differences between groups. The impact coefficient was also calculated for each group. Data analysis at the significance level P<0.05 and was performed using SPSS software version 26. |
| --- |

**Research background**

| Ali Arnejad and Magellan (1400)(49) In a study comparing the effects of an eight-week training program with two different strengthening and stretching patterns on changes in the status of lower crossed syndrome, 45 students diagnosed with lumbar lordosis and a curvature angle of more than 45 degrees were randomly assigned to three groups of fifteen. Group one performed exercises with time intervals, group two performed exercises simultaneously, and group three was the control group.  After eight weeks of training, the findings indicated a significant difference between the interval training and the control group, but no significant difference between the simultaneous training and the control group. As a result, based on the findings of this study, performing stretching and strengthening exercises with intervals has a greater effect on reducing lumbar lordosis than performing these exercises simultaneously. (49).  Research by Badihi and Mahdavinejad (2019)(48) examined the effect of eight weeks of selected corrective exercises (NASM) on correcting lumbar lordosis and improving the balance of female karate athletes in Isfahan. In this study, 40 athletes participated as subjects in two control and experimental groups. The lumbar lordosis angle, static balance, and dynamic balance of the subjects were evaluated before and after the exercises. The results indicated that NASM exercises caused a significant reduction in the lumbar lordosis angle and improved static balance, as well as an increase in the reach distance in dynamic balance. (48).  In a study conducted by Abdollahzadeh and Daneshmandi (2019)(10), the effect of eight weeks of corrective exercises based on the principles of NASM was investigated on upper crossed syndrome. Thirty female students with a head forward angle of more than 46 degrees, shoulder forward angle of more than 52 degrees, and kyphosis of more than 42 degrees were purposefully selected and then randomly divided into two control and experimental groups. Subjects in the experimental group performed corrective exercises for eight weeks. The results showed that eight weeks of corrective exercises based on NASM principles led to improvements in head forward, shoulder forward, and kyphosis angles in the experimental group compared to the control group. (10).  Okhli et al. (2018) conducted a study to compare the effects of corrective exercises from the National Academy of Sports Medicine (NASM) and Pilates on the correction of lordosis in female high school students. In this experimental study, 45 female high school students with lumbar lordosis were randomly selected and divided into three groups: NASM corrective exercises, Pilates exercises, and control. The two intervention groups received corrective exercises for eight weeks. According to the results, the lumbar lordosis angle decreased in both NASM and Pilates exercise groups following the intervention, with NASM exercises leading to a greater reduction in lumbar curvature compared to Pilates exercises. (34).  The purpose of the study by Kamali et al. (2015)(50) was to examine the effect of eight weeks of NASM training on lumbar lordosis and some biomotor skills of female students. In this quasi-experimental study, 30 students with a curvature angle of more than 30 degrees were selected and randomly assigned to two groups: NASM exercises and control. The results showed that NASM exercises reduced lumbar lordosis and significantly increased the strength and endurance of the core muscles. Their research also indicated a significant increase in the flexibility of the back muscles and improved balance. (50).  Ganji and Tahmorsi (2015)(51) conducted a study aimed at investigating the effect of corrective exercises and posture training on some indicators of lower back syndrome. Forty students with lower crossed syndrome participated in corrective exercises in two groups for 10 weeks. The lordosis angle and pelvic tilt were lower in the group that received both corrective exercises and postural training than in the group that received corrective exercises alone. Members of the combined group also exhibited greater abdominal muscle strength compared to the corrective exercise group. However, there was no significant difference between the groups in terms of increasing flexibility in the hamstring, rectus femoris, adductor, and spinal erector muscles. (51).  Khan et al. (2022) compared the effects of stretching and muscle energy techniques in the treatment of lower crossed syndrome. In this parallel study, 58 individuals with lower crossed syndrome were randomly assigned to two intervention groups. Group A received stretching techniques, and Group B received muscle energy techniques, three sessions per week for a total of four weeks. This study concluded that there was a statistically significant difference in the variables of both groups, namely the numerical pain rating scale (NPRS), Oswestry Disability Index (ODI), bilateral Suez muscle length, hamstrings, rectus femoris, and spinal erectors. However, the mean values of the above parameters showed a slight improvement in the muscle energy technique group. ^[[28]](#footnote-28)^^[[29]](#footnote-29)^  The purpose of the study by Sahu and Phansopkar (2021) was to screen for low back syndrome in asymptomatic individuals. Three hundred men and women participated in this study. The REEDCO Posture Scale was used for the initial posture examination. The length of the bilateral rectus abdominis muscle was measured using a goniometer and a modified Thomas test. A non-elastic measuring tape was used to examine the length of the erector spinae muscles. The strength of the bilateral abdominal and gluteus maximus muscles was graded using a manual muscle test based on the MRC grading system. According to the findings, women exhibited more muscle tension and weakness, especially in the rectus abdominis muscle. There were also significant differences in the length of the erector spinae, transversus abdominis, and gluteus maximus muscles. This study also showed that women were more likely to suffer from low back syndrome than men.  Adam (2014) In their study, they examined the effect of muscle energy technique versus chiropractic intervention in the treatment of chronic low back pain in people with lower cross syndrome. Thirty participants were divided into three groups (muscle energy technique and chiropractic intervention group) and a combined group (muscle energy technique and chiropractic interventions). Numerical pain rating scale, Oswestry functional disability questionnaire, active range of motion and lordosis angle were evaluated in these individuals. The results of this study showed that chiropractic interventions, muscle energy technique and a combination of muscle energy technique and chiropractic interventions are effective treatment protocols in reducing pain and disability and have the ability to restore proper mechanical function and, as a result, increase lumbar range. They also reduce the degree of lumbar lordosis. On the other hand, there was no treatment protocol that was more effective than the other. |
| --- |

**Executive application**

| Implementing an effective exercise program for people with lower crossed syndrome, coaches, corrective exercise specialists, and therapists on Lordosis angle, spinal range of motion, core muscle endurance, and selected muscle activity. |
| --- |

**Scheduling the work steps**

| **Time/ Month**  **Implementation steps** | **Month** | | | | | | | | | | | |
| --- | --- | --- | --- | --- | --- | --- | --- | --- | --- | --- | --- | --- |
|  | **1** | **2** | **3** | **4** | **5** | **6** | **7** | **8** | **9** | **10** | **11** | **12** |
| Research and proposal writing |  |  |  |  |  |  |  |  |  |  |  |  |
| Pre-test data collection |  |  |  |  |  |  |  |  |  |  |  |  |
| Research Executive Department |  |  |  |  |  |  |  |  |  |  |  |  |
| Data collection after we |  |  |  |  |  |  |  |  |  |  |  |  |
| Data analysis |  |  |  |  |  |  |  |  |  |  |  |  |
| Thesis writing |  |  |  |  |  |  |  |  |  |  |  |  |

**List of references (in order of use in the text, in Vancouver format and with EndNote software)**

| 1. Ch A, Shams Majalan A. Comparison of the Effect of Eight Week Training Program Schedule With Two Different Stretching Patterns Strengthening on Changes in the Status of Lower Cross Syndrome. Journal of Sport Biomechanics. 2021;7(1):108-21.  2. Rajabi R, Seidi F, Mohamadi F. Which method is accurate when using the flexible ruler to measure the lumbar curvature angle? Deep point or mid-point of arch. World Appli Sci. 2008;4(6):849-52.  3. Gültekin H, Bayram D, Yüksel GA, Bayram T, Tireli H. Assessment of Modified-Modified Schober Test and Lumbar Range of Motion in Patients with Parkinson's Disease with and Without Low Back Pain. Turkish Journal of Neurology/Turk Noroloji Dergisi. 2022;28(1).  4. De Luca CJ. The use of surface electromyography in biomechanics. Journal of applied biomechanics. 1997;13(2):135-63.  5. Key J. The Pelvic Crossed Syndromes: A reflection of unbalanced function in the myofascial envelope; a further exploration of Janda's work. Journal of bodywork and movement therapies. 2010;14(3):299-301.  6. Janda V, Jull G. Muscles and motor control in low back pain: assessment and management. Physical Therapy of the Low Back. T Twomey, Churchill Livingstone New York p253-78. 1987.  7. Ishida H, Hirose R, Watanabe S. Comparison of changes in the contraction of the lateral abdominal muscles between the abdominal drawing-in maneuver and breath held at the maximum expiratory level. Manual therapy. 2012;17(5):427-31.  8. Zaprawa K, Filipowicz P. Lower crossed syndrome (LSD). Adv Sci Med. 2018;3(1):5-13.  9. Das S, Sarkar B, Sharma R, Mondal M, Kumar P, Sahay P. Prevalence of lower crossed syndrome in young adults: A cross sectional study. Int J Adv Res. 2017;5(6):2217-28.  10. Abdolahzadeh M, Daneshmandi H. The Effect of an 8-week NASM Corrective Exercise Program on Upper Crossed Syndrome. Journal of Sport Biomechanics. 2019;5(3):156-67.  11. Sahu P, Phansopkar P. Screening for lower cross syndrome in asymptomatic individuals. J Med Pharm Allied Sci. 2021;10(6):8-3894.  12. Dhanani S, Shah DT. A survey on the prevalence of lower crossed syndrome in young females. IJPSH. 2014;1:2249-5738.  13. Burton AK, Tillotson K. Is recurrent low back trouble associated with increased lumbar sagittal mobility? Journal of Biomedical Engineering. 1989;11(3):245-8.  14. Jones G, MacFarlane GJ. Epidemiology of low back pain in children and adolescents. Archives of diseases in childhood. 2005;90(3):6-312.  15. Rahimi M, Sadeghiyan M, Samadi H. The six weeks effect of selected core stabilization exercises on lumbar curvature and functional movement screening test in women with lower crossed syndrome in Covid Pandemic 19. Anesthesiology and Pain. 2022;13(3):96-108.  16. Mandarakas MR, Young P, Burns J. Neuromuscular rehabilitation–what to do? Current Opinion in Neurology. 2021;34(5):697-705.  17.Yarahmadi Y, Mirbaghri R, Hdadnzhad M. Efficacy of the Yumeiho Therapy Massage on Repositioning Error, Range of Motion of Trunk Flexion and the Functional Power in Female Volleyball Players with Hyper Lordosis. Journal of Sport Biomechanics. 2018;3(4):51-61.  18.Lei T, Tong T, Miao D, Gao X, Xu J, Zhang D, et al. Anterior migration after Bryan cervical disc arthroplasty: the relationship between hyperlordosis and its impact on clinical outcomes. World neurosurgery. 2017;101:534-9.  19.do Rosário JLP, Nakashima IY, Rizopoulos K, Kostopoulos D, Marques AP. Improving posture: comparing segmental stretch and muscular chain therapy. Clinical Chiropractic. 2012;15(3-4):121-8.  20.Kendall FP, McCreary EK, Provance PG, Rodgers MM, Romani WA. Muscles: testing and function with posture and pain: Lippincott Williams & Wilkins Baltimore, MD; 2005.  21.Capson AC, Nashed J, Mclean L. The role of lumbopelvic posture in pelvic floor muscle activation in continent women. Journal of Electromyography and Kinesiology. 2011;21(1):166-77.  22.Liebenson C. Rehabilitation of the spine: a practitioner's manual: Lippincott Williams & Wilkins; 2007.  23.Latalski M, Bylina J, Fatyga M, Repko M, Filipovic M, Jarosz MJ, et al. Risk factors of postural defects in children at school age. Annals of agricultural and environmental medicine. 2013;20(3).  24.Izraelski J. Assessment and treatment of muscle imbalance: The Janda approach. The Journal of the Canadian Chiropractic Association. 2012;56(2):158.  25.Claeys K, Brumagne S, Deklerck J, Vanderhaeghen J, Dankaerts W. Sagittal evaluation of usual standing and sitting spinal posture. Journal of bodywork and movement therapies. 2016;20(2):326-33.  26.Abbaszadeh A, Sahebzamani M, Seifadini M, Samsampour D. Effect of an 8 week corrective exercise on hyperlordosis female students, Kerman, Iran. Hormozgan Med J. 2012;16(5):377-86.  27.Golestani N, Seidi F, Minoonejad H. Comparison of Lower Extremity Function in Non-Athlete Females with and without Lumbar Hyper Lordosis. The Scientific Journal of Rehabilitation Medicine. 2019;8(2):56-66.  28.Wilson JD, Dougherty CP, Ireland ML, Davis IM. Core stability and its relationship to lower extremity function and injury. JAAOS-Journal of the American Academy of Orthopedic Surgeons. 2005;13(5):316-25.  29.Tse MA, McManus AM, Masters RS. Development and validation of a core endurance intervention program: implications for performance in college-age rowers. The Journal of Strength & Conditioning Research. 2005;19(3):547-52.  30.Sadler SG, Spink MJ, Ho A, De Jonge XJ, Chuter VH. Restriction in lateral bending range of motion, lumbar lordosis, and hamstring flexibility predicts the development of low back pain: a systematic review of prospective cohort studies. BMC Musculoskeletal Disorders. 2017;18(1):179.  31.Vigotsky AD, Lehman GJ, Beardsley C, Contreras B, Chung B, Feser EH. The modified Thomas test is not a valid measure of hip extension unless pelvic tilt is controlled. PeerJ. 2016;4:e2325.  32.Arab AM, Haghighat A, Amiri Z, Khosravi F. Lumbar lordosis in prone position and prone hip extension test: comparison between subjects with and without low back pain. Chiropractic & Manual Therapies. 2017;25(1):8.  33.Lehman GJ, Lennon D, Tresidder B, Rayfield B, Poschar M. Muscle recruitment patterns during the prone leg extension. BMC Musculoskeletal Disord. 2004;5:3.  34.Okhli H, Hojjati H, Akhoundzadeh G. Comparing the Effect of the Corrective Exercises of America's National Academy of Sports Medicine and Pilates on the Correction of Lordosis among Female High School Students in Golestan Province in 2018. International Journal of School Health. 2019;6(4):1-6.  35.Clark M, Luckett S. NASM essentials of corrective exercise training: Lippincott Williams & Wilkins; 2010.  36.Norasteh A, Hajihosseini E, Emami S, Mahmoudi H. Assessing Thoracic and Lumbar Spinal Curvature Norm: A Systematic Review. Physical Treatments - Specific Physical Therapy. 2019;9(4):183-92.  37.Youdas JW, Hollman JH, Krause DA. The effects of gender, age, and body mass index on standing lumbar curvature in persons without current low back pain. Physiotherapy theory and practice. 2006;22(5):229-37.  38. Gonzalez-Galvez N, Gea-Garcia GM, Marcos-Pardo PJ. Effects of exercise programs on kyphosis and lordosis angle: A systematic review and meta-analysis. PloS one. 2019;14(4):e0216180.  39. Nabavi N, MOHSENI BM, Mosallanejad Z, Rahgozar M. Reliability of measuring lumbar range of motion using modified-modified Schober test in healthy subjects. 2011.  40. Rezvani A, Ergin O, Karacan I, Oncu M. Validity and reliability of the metric measurements in the assessment of lumbar spine motion in patients with ankylosing spondylitis. Spine. 2012;37(19):E1189-E96.  41. Saki F, Ramezani F. Comparison of Range of Motion and Strength of Hip Muscles in Female Athletes with and without Dynamic Knee Valgus. Avicenna Journal of Clinical Medicine. 2020;27(2):9-100.  42. Kiseljak D, Medved V. Motor recruitment pattern during the prone hip extension test: is hip extension initiated by the hip or the lumbar extensor muscles? International Journal of Therapy and Rehabilitation. 2022;29(5):1-11.  43. Krause DA, Hollman JH. Electromyographic Analysis of Hip Muscle Activation during a Single Limb Squat Lateral Slide Exercise. International Journal of Sports Physical Therapy. 2020;15(5):755.  44. Cuesta-Vargas AI, González-Sánchez M. Differences in muscle activation patterns during sit to stand task among subjects with and without intellectual disability. BioMed research international. 2013; 2013.  45. O'Sullivan K, Smith SM, Sainsbury D. Electromyographic analysis of the three subdivisions of gluteus medius during weight-bearing exercises. BMC Sports Science, Medicine and Rehabilitation. 2010;2(1):1-9.  46. Macadam P, Feser EH. Examination of gluteus maximus electromyographic excitation associated with dynamic hip extension during body weight exercise: a systematic review. International journal of sports physical therapy. 2019;14(1):14.  47. Hodges PW, Bui BH. A comparison of computer-based methods for the determination of onset of muscle contraction using electromyography. Electroencephalography and Clinical Neurophysiology/Electromyography and Motor Control. 1996;101(6):511-9.  48. Mahdavinejad R, Badihi M. Effects of 8-week selective corrective exercises program on the correction of lumbar lordosis and improving the balance in female karate athletes in Isfahan. Razi Journal of Medical Sciences. 2020;27(10):50-62.  49. Alyarnezhad C, Shams Majalan A. Comparison of the Effect of Eight Week Training Program Schedule With Two Different Stretching Patterns Strengthening on Changes in the Status of Lower Cross Syndrome. Journal of Sport Biomechanics. 2021;7(2):108-21.  50. Kamali M, Ghasemi B, BagherianDehkordi S. Effect of 8-week NASM's corrective exercise continuum on correction of lumbar lordosis and some biomotor skills in female students with hyperlordosis. Journal for Research in Sports Rehabilitation. 2015;3(5):31-41.  51. Ganji B, Tahmouresi L. Effect of corrective exercises and educating posture on some lower cross syndrome indices. The 8th National Conference on Sport Sciences and Physical Education of Iran: Sport research institute of Iran; 2015.  52. Esakowitz A. The Effect of Muscle Energy Technique Versus Chiropractic Adjustive Therapy in the Treatment of Chronic Low Back Pain with Lower Cross Syndrome: University of Johannesburg (South Africa); 2014. |
| --- |

1. Muscle imbalance [↑](#footnote-ref-1)
2. Lower Cross Syndrome [↑](#footnote-ref-2)
3. Muscle imbalance [↑](#footnote-ref-3)
4. Sagittal plane [↑](#footnote-ref-4)
5. Lumbo-pelvic movement [↑](#footnote-ref-5)
6. Rectus abdominis [↑](#footnote-ref-6)
7. Gluteus maximus [↑](#footnote-ref-7)
8. Gluteus medius [↑](#footnote-ref-8)
9. Gluteus minimus [↑](#footnote-ref-9)
10. Erector spinae [↑](#footnote-ref-10)
11. Multifidus [↑](#footnote-ref-11)
12. Quadratus lumborum [↑](#footnote-ref-12)
13. Latissimus dorsi [↑](#footnote-ref-13)
14. Iliopsoas [↑](#footnote-ref-14)
15. Tensor fasciae latae. [↑](#footnote-ref-15)
16. Sacroiliac joint [↑](#footnote-ref-16)
17. Prone hip extension [↑](#footnote-ref-17)
18. National Academy of Sports Medicine [↑](#footnote-ref-18)
19. Myofascial release [↑](#footnote-ref-19)
20. Body Mass Index [↑](#footnote-ref-20)
21. Thoracic [↑](#footnote-ref-21)
22. [↑](#footnote-ref-22)
23. Modified - Modified Schober's Test [↑](#footnote-ref-23)
24. Anterior Superior Iliac Spine [↑](#footnote-ref-24)
25. Longissimus muscle [↑](#footnote-ref-25)
26. Biceps Femoris [↑](#footnote-ref-26)
27. Maximal Voluntary Isometric Contraction (MVIC) [↑](#footnote-ref-27)
28. Numerical Pain Rating Scale [↑](#footnote-ref-28)
29. Oswestry Disability Index [↑](#footnote-ref-29)
