# Supplementary material for: The effect of NASM-based corrective exercises on lumbar lordosis angle and selected muscle activity in women with lower cross syndrome: A randomized clinical trial
Source: PLoS One. 2026 Mar 4;21(3):e0337804. doi: 10.1371/journal.pone.0337804 (PMC12959714; doi:10.1371/journal.pone.0337804)
Supplement: Attachment — Submitted filename: Response to Reviewers.docx [file pone.0337804.s005.docx]

Dear Editor and Reviewers,

Thank you very much for allowing us to revise our manuscript [PONE-D-25-25748] entitled "The effect of NASM-based corrective exercises on lumbar lordosis angle and selected muscle activity in women with lower cross syndrome: A randomized clinical trial". We sincerely appreciate the time and effort you have dedicated to providing insightful comments and valuable suggestions. We have carefully considered all the points raised and have made significant revisions to the manuscript accordingly.

Please note that all line numbers referenced in our point-by-point responses below correspond to the Revised Manuscript with Track Changes file.

Our detailed responses to each comment are provided below.

Journal Requirements:

Comment 1: Please ensure that your manuscript meets PLOS ONE's style requirements, including those for file naming.

Response: We confirm that the revised manuscript has been formatted to comply with PLOS ONE's style requirements.

Comment 2: We note that you have selected “Clinical Trial” as your article type. PLOS ONE requires that all clinical trials are registered in an appropriate registry.

Response: The study was registered in the Iranian Registry of Clinical Trials (IRCT) with the code IRCT20240805062660N1. This registration number and the date range for participant recruitment (March 1 to May 31, 2024) have been added to the manuscript's title page and Methods section, respectively.

Comment 3: PLOS requires an ORCID ID for the corresponding author in Editorial Manager on papers submitted after December 6th, 2016.

Response: The corresponding author's ORCID ID has been validated in the Editorial Manager system.

Comment 4: Please ensure that you clearly indicate the corresponding author in the title page of the manuscript.

Response: The corresponding author is clearly indicated with an asterisk (*) on the title page of the manuscript.

Comment 5: Your ethics statement should only appear in the Methods section of your manuscript.

Response: The ethics statement has been moved solely to the Methods section (Method of Execution) and removed from the standalone section.

Comment 6: We note that Figures 2, 3, and SI Files (Corrective Exercise Protocol NASM.docx, Original_Protocol_English_Translation.docx) include images of participants in the study.

Response: We confirm that written informed consent for publication has been obtained from the individuals depicted in Figures 2, 3, and SI files as outlined in the PLOS consent form. A statement to this effect has been added to the Methods section.

Comment 7: Please include captions for your Supporting Information files at the end of your manuscript, and update any in-text citations to match accordingly.

Response: A "Supporting Information" section has been added to the end of the manuscript, providing captions for all supplementary files.

Comment 8: We note that the original protocol that you have uploaded as a Supporting Information file contains an institutional logo.

Response: The institutional logo has been removed from the supporting information file, and an updated version has been uploaded.

Reviewer #1:

Comment 1: Do the exclusion criteria include any drug, tobacco or alcohol use?

Response: We appreciate the reviewer's important point. Our exclusion criteria did not initially address substance use. We have now revised the exclusion criteria in the "Population and Sampling" section to include: "Regular use of tobacco, alcohol, or any medication that could affect neuromuscular function or pain perception." (Lines 159-160)

Comment 2: What does EA mean? Please indicate a significance level of p≤0.05 below the tables.

Response: We apologize for this oversight. We have clarified that "EA" stands for "Electrical Activity" (normalized as a percentage of MVIC) in the footnotes of all Tables. Furthermore, we have added the note "Significance level was set at p ≤ 0.05" below the tables. (Lines below Tables 1, 2, and 3)

Comment 3: The Discussion section should be improved. Please evaluate the strengths and weaknesses of the study.

Response: We sincerely thank the reviewer for this constructive suggestion. We have thoroughly revised the Discussion section to provide a deeper exploration of the meaning and implications of our findings. Most importantly, we have added a new dedicated paragraph at the end of the Discussion to state the strengths and limitations of our study. (Lines 352-407)

Reviewer #2:

Comment 1: In the power analysis, the effect size was assumed to be 0.5 without any justification.

Response: We thank the reviewer for highlighting this. The effect size of 0.5 was selected based on similar previous studies investigating the effect of corrective exercises on lumbar lordosis and muscle activity, which reported medium to large effect sizes. We have now added this justification to the "Population and Sampling" section: "...an effect size of 0.5 (representing a medium effect, based on similar studies on LCS [12,14]) ..." (Line 147)

Comment 2: Please comment on the inter-rater or intra-rater reliability for the measures. Response: This is a valuable point. We have added a statement regarding the reliability of our primary outcome measure. In the "Lumbar Lordosis Angle Measurement" subsection, we now state: "To ensure consistency, all measurements were performed by the same experienced researcher, using a method with high reported reliability (ICC = 0.97) [13]." (Lines 179-181) For the EMG, the standardized SENIAM protocol and electrode placement, conducted by a single researcher, ensured measurement consistency. (Lines 185-186)

Comment 3: It would be informative to know whether the pre-test values differ between the two groups.
Response: We sincerely thank the reviewer for this valuable suggestion. We have now conducted comprehensive independent samples t-tests to compare all baseline outcome variables between the control and exercise groups. These results have been incorporated into the revised manuscript in two key ways:

1. A new comprehensive Table 1 has been created, titled "Baseline Characteristics and Clinical Outcomes of the Study Participants". This table presents the pre-test means and standard deviations for all demographic and outcome variables (including lumbar lordosis angle and all EMG parameters) for both groups, along with the p-values from the between-group comparisons.
2. The Results section has been updated to state: "The independent t-test results for participants' demographic characteristics and all baseline outcome variables are presented in Table 1. The groups were homogeneous in terms of age, weight, and height (p > 0.05). However, baseline comparisons revealed significant differences between the groups in the pre-test values of EA Hamstrings (p=0.003), MVIC Hamstrings (p=0.029), and MVIC Erector Spinae (p=0.017). These baseline variables were therefore included as covariates in their respective ANCOVA models to control for pre-existing differences." (Lines 257-262)

This analysis confirmed that the groups were comparable at baseline for most variables, but it also identified the specific pre-existing differences mentioned above. We believe this addition significantly strengthens the manuscript by providing a complete picture of baseline equivalence and justifying our use of ANCOVA for the primary analysis.

Comment 4: Are there any units for the variables in Table 2?

Response: We thank the reviewer for this important observation. We have now added the respective units directly after each variable name in all Tables of the revised manuscript to ensure immediate clarity and ease of interpretation for the reader. (Lines Tables 1, 2, and 3)

Comment 5: Please clarify what is presented in Table 3.

Response: We thank the reviewer for this question. We have clarified the presentation in the revised manuscript. The values in Table 3 ("Between-group Comparison of Adjusted Post-Test Outcomes Using ANCOVA") represent the "Adjusted Post-test Means".

To ensure statistical rigor, we used ANCOVA for all outcome variables, controlling for their respective pre-test scores as covariates. Therefore, the values 8.83 and 10.47 represent the statistically adjusted mean post-intervention electrical activity of the hamstrings (%MVIC) for the control and exercise groups, respectively, after controlling for their baseline values. This method provides a more precise estimate of the intervention effect by adjusting for any pre-existing variation.

Reviewer #3:

Comment 1: The abstract should specify the exact measurement tools more clearly.

Response: We thank the reviewer for this suggestion. We have revised the Methods section of the abstract (Lines 36-39) to specify the measurement tools as follows:

- Lumbar lordosis: 30 cm flexible ruler (KEARING brand, China)
- Muscle activity: Myon 320 surface electromyography system (Switzerland) during maximum voluntary isometric contraction

This clarification provides readers with a clearer understanding of our measurement methodology from the very beginning of the article.

Comment 2: Clarify the abstract results to distinguish between-group and within-group changes.
Response: We have carefully reworded the results in the abstract to avoid confusion. It now clearly states the significant between-group differences for lumbar lordosis and gluteus maximus activity, and separately mentions the significant within-group changes observed in the exercise group for other variables. (Lines 51-59)

Comment 3: Clarify the randomization process and allocation concealment.

Response: We have added more detail to the "Population and Sampling" section: "Using a computer-based random number generator, an independent researcher who was not involved in recruitment or assessment allocated participants to either the experimental or control group. The allocation sequence was concealed until groups were assigned." (Lines 149-152)

Comment 4: The novelty of the study is not clearly stated.

Response: We have strengthened the final paragraph of the Introduction to explicitly state the novelty: "While the effect of NASM exercises on lumbar lordosis is becoming established, the literature lacks comprehensive evidence regarding their impact on the electromyographic activity of the entire posterior kinetic chain in individuals with LCS. In particular, the response of the hamstrings to this specific intervention remains relatively unexplored, despite their crucial role in pelvic dynamics and their known involvement in compensatory patterns within LCS…" (Lines 116-129)

Comment 5: Provide exact p-values for all non-significant results in Table 2 and Table 3.

Response: We thank the reviewer for this suggestion, which enhances transparency. We have replaced all instances of "p>0.05" in all Tables with the exact p-values. (Lines in Tables 1, 2, and 3)

Comment 6: Clarify EMG processing details (filtering, sampling frequency, software).

Response: This is a crucial point for reproducibility. We have added the following details to the "Maximum Voluntary Isometric Contraction (MVIC)" subsection: "The raw EMG signals were sampled at 2000 Hz. Signal processing was performed using MATLAB software (MathWorks, USA). The raw data were first band-pass filtered (20-500 Hz), and a 50 Hz notch filter was applied to remove mains electricity interference." (Lines 188-191)

Comment 7: Replace non-standard terms: “semi-experimental randomized design”.

Response: We have replaced the term "semi-experimental" throughout the manuscript with the more standard term "randomized controlled trial". (For example, in the Abstract line 32, and the Methodology section lines 131 and 211)

Comment 8: Remove unsupported claims and overgeneralizations in the conclusion (e.g., “improving range of motion”, “distribution of forces”).

Response: We agree and have revised the Conclusion to strictly reflect our findings. The unsupported claims about "range of motion" and "distribution of forces" have been removed. The conclusion now focuses directly on the outcomes we measured. (Lines 414-427)

Comment 9: Make the research gap explicit regarding hamstring activity.

Response: As mentioned in response to comment #4, we have explicitly highlighted the gap concerning hamstring activity in the Introduction. (Lines 116-129)

Comment 10: Address unexpected results in the control group (e.g., significant changes in hamstring activity).

Response: This is an excellent observation. We have added a sentence to the Discussion to address this: "The unexpected significant change in hamstring electrical activity within the control group may be attributed to measurement variability, unaccounted daily activities, or habituation to testing procedures, highlighting the importance of using a controlled design to isolate the specific effect of the intervention." (Lines 387-390)

Comment 11: Rename tables with clear, self-explanatory titles.

Response: We have revised all table titles as suggested:

- Table 1 is now: "Baseline Characteristics and Clinical Outcomes of the Study Participants"
- Table 2 is now: "Within-group Pre- and Post-test Comparisons of Lumbar Lordosis Angle and Muscle Activity"
- Table 3 is now: "Between-group Comparison of Adjusted Post-Test Outcomes using ANCOVA"
  (Title Tables 1, 2, and 3)

Comment 12: Provide representative continuous EMG traces.

Response: We thank the reviewer for this suggestion. We have now included a new supplementary figure (S1 Fig) showing representative filtered raw EMG traces for all three muscles during the MVIC task, both pre- and post-intervention, for a participant from the exercise group. A caption for this file has been added to the new "Supporting Information" section. (See Supporting Information section)

Comment 13: Figure 2 shows a motion capture camera, but no system is described.

Response: We apologize for this oversight. The camera was not part of the data collection and was present in the lab for other purposes. To avoid confusion, we have replaced Figure 2 with a new, clearer image that focuses solely on the electrode placements without any extraneous equipment in the background.

Comment 14: Differentiate between the study’s novel findings and previously reported results.

Response: We have revised the Discussion to make this distinction clearer. We now explicitly state: "The reduction in lumbar lordosis (F = 24.82, p < 0.001) aligns with previous research employing the NASM approach … (Lines 361-376). A novel and clinically significant finding was the robust between-group improvement in gluteus maximus electrical activity …" (Lines 377-383)

Comment 15: Consider recommending future research rather than new interventions.

Response: We have rephrased the relevant sentence in the Discussion as a recommendation for future research: "Future studies should investigate the long-term effects of NASM exercises, include a more diverse population, and explore the effects of supplementing the protocol with targeted exercises for the hamstrings and erector spinae to elicit more comprehensive neuromuscular adaptations." (Lines 404-407)

Comment 16: Condense and focus the conclusion. Do not introduce new claims.

Response: We have entirely rewritten the Conclusion to be more focused and specific. It now concisely summarizes the key findings, their direct implication, and avoids unsupported claims about quality of life or broad abnormalities. (Lines 414-427)

Comment 17: Restructure the conclusion to include limitations and future research.

Response: As suggested, we have expanded the Conclusion into a more structured paragraph that includes: a summary of key findings, the main clinical implication, the primary study limitations (e.g., sample demographics), and a brief suggestion for future research. (Lines 414-427)
