# Supplementary material for: The effect of NASM-based corrective exercises on lumbar lordosis angle and selected muscle activity in women with lower cross syndrome: A randomized clinical trial
Source: PLoS One. 2026 Mar 4;21(3):e0337804. doi: 10.1371/journal.pone.0337804 (PMC12959714; doi:10.1371/journal.pone.0337804)
Supplement: Attachment — Submitted filename: Response_to_Reviewers_auresp_2.docx [file pone.0337804.s006.docx]

**Manuscript ID:** PONE-D-25-25748R1

**Title:** The effect of NASM-based corrective exercises on lumbar lordosis angle and selected muscle activity in women with lower cross syndrome: A randomized clinical trial

We would like to express our sincere gratitude to the Academic Editor and the reviewers for their valuable time and insightful comments on our manuscript. Their constructive feedback has been instrumental in helping us improve the quality and clarity of our work. We have carefully considered all points raised and have revised the manuscript accordingly. Our point-by-point responses are detailed below.

All changes made in the manuscript have been highlighted in the "Revised Manuscript with Track Changes" file for the reviewers' convenience.

**Comments from Academic Editor:**

We thank the Academic Editor for overseeing the review process and for the opportunity to revise our manuscript.

**Comments from Reviewer #1:**

**Comment 1:** "When the discussion section is considered, it is observed that the study exhibits certain limitations. A comprehensive discussion should ideally include a detailed comparison of the study's findings with those of previous studies to highlight similarities, differences, and potential reasons for discrepancies. Such comparisons are essential for situating the research within the broader scientific discourse, thereby enhancing its credibility and relevance."

**Response:** We sincerely thank the reviewer for this crucial comment. We completely agree that a comprehensive discussion strengthens the manuscript. In response, we have thoroughly revised and expanded the Discussion section to provide a detailed comparison of our findings with those of previous, relevant studies.

Specifically, we have now:

- Directly compared our findings on lumbar lordosis reduction with the results of Ghadirian Marnani et al. (2024) and Okhli et al. (2019), highlighting the consistency of the NASM protocol's effectiveness. (Lines 262-268)
- Contextualized our novel EMG finding on gluteus maximus activity by comparing it with the functional improvements reported by Samadi and Hajilo (2024) and emphasized the added value of our objective EMG data. (Lines 285-289)
- Addressed the non-significant between-group result for hamstring activity by contrasting it with the compensatory role of hamstrings described by Lehman et al. (2004), offering a methodological explanation (reduced compensatory demand due to targeted gluteal activation) for the discrepancy. (Lines 297-303)
- Discussed our within-group finding on erector spinae activation timing in the context of the work by Kim et al. (2014). (Lines 310-311)

These additions ensure our results are critically situated within the existing scientific literature, as the reviewer recommended.

**Comment 2:** "Furthermore, a well-rounded discussion should consider methodological differences, sample characteristics, and contextual factors that could influence the results. By doing so, the study can provide a more nuanced interpretation of its findings and establish a clearer link with existing knowledge."

**Response:** We thank the reviewer for this excellent suggestion, which has helped us provide a more nuanced interpretation. In the revised Discussion, we have explicitly addressed these factors:

- Methodological Differences: We now discuss how the specific focus of our NASM protocol (on gluteal activation) might explain the difference in hamstring activity findings compared to other studies that merely described the compensatory pattern (e.g., Lehman et al., 2004). (Lines 300-303)
- Sample Characteristics: We have reinforced the description of our sample (young women) when comparing our results with studies on similar populations (e.g., Okhli et al., 2019) (Lines 264-267). Furthermore, we have retained and emphasized the limitation regarding the generalizability of our findings beyond young women in the 'Limitations' paragraph. (317-319)
- Contextual Factors / Nuanced Interpretation: We have moved beyond simply reporting results to interpreting them through potential physiological mechanisms. For example, we propose that the improved gluteal function might have reduced the compensatory load on the hamstrings, offering a plausible reason for their less pronounced change in the between-group analysis. This provides a more mechanistic and nuanced understanding of our results. (300-303)

We believe these revisions have significantly strengthened the discussion by creating clearer links with existing knowledge and offering deeper insights.

**Comments from Reviewer #2:**

(No Response)

Response: We thank Reviewer #2 for their time and consideration of our manuscript.

**Comments from Reviewer #3:**

"Every point has been explicitly addressed in revised version, including clarification of the abstract, detailed EMS information, a clearer novelty statement, improved tables, restructured discussion, and a more focused conclusion. Thank you for the thorough revision."

**Response:** We are deeply grateful to Reviewer #3 for their positive and encouraging feedback and for acknowledging the thoroughness of our previous revisions. We are pleased that the reviewer finds the manuscript substantially improved.

**Conclusion**

Once again, we extend our heartfelt thanks to the editor and the reviewers for their constructive comments, which have undoubtedly improved the quality of our manuscript. We hope that our revisions and responses are now satisfactory and that the manuscript is deemed suitable for publication in PLOS ONE.

Sincerely,

Seyed Mohammad Hosseini, Ph.D.

On behalf of all co-authors.
